# Nuclear Ssr4 Is Required for the *In Vitro* and *In Vivo* Asexual Cycles and Global Gene Activity of Beauveria bassiana

**DOI:** 10.1128/mSystems.00677-19

**Published:** 2020-04-21

**Authors:** Wei Shao, Qing Cai, Sen-Miao Tong, Sheng-Hua Ying, Ming-Guang Feng

**Affiliations:** a MOE Laboratory of Biosystems Homeostasis & Protection, College of Life Sciences, Zhejiang University, Hangzhou, Zhejiang, China; b College of Agricultural and Food Science, Zhejiang A&F University, Lin’an, Zhejiang, China; USDA Agricultural Research Service, Boyce Thompson Institute, Cornell University

**Keywords:** virulence, aerial conidiation, chromatin-remodeling SWI/SNF subunit, entomopathogenic fungi, gene expression and regulation, hyphal differentiation, hyphal hydrophobicity, insect pathogenicity

## Abstract

Ssr4 serves as a cosubunit of chromatin-remodeling SWI/SNF and RSC complexes in yeasts but remains functionally uncharacterized due to its essentiality for yeast viability. Here, we report pleiotropic effects of the deletion of the *ssr4* ortholog nonessential for cell viability in Beauveria bassiana, an asexual insect mycopathogen. The deletion of *ssr4* resulted in severe growth defects on different carbon/nitrogen sources, increased hyphal hydrophilicity, blocked hyphal differentiation, and 98% reduced conidiation capacity compared to a wild-type standard. The limited Δ*ssr4* conidia featured an impaired coat with disordered or obscure hydrophobin rodlet bundles, decreased hydrophobicity, increased size, and lost insect pathogenicity via normal cuticle infection and 90% of virulence via intrahemocoel injection. The expression of genes required for hydrophobin biosynthesis and assembly of the rodlet layer was drastically repressed in more hydrophilic Δ*ssr4* cells. Transcriptomic analysis revealed 2,517 genes differentially expressed in the Δ*ssr4* mutant, including 1,505 downregulated genes and 1,012 upregulated genes. The proteins encoded by hundreds of repressed genes were involved in metabolism and/or transport of carbohydrates, amino acids, and lipids, inorganic ion transport and energy production or conversion, including dozens involved in DNA replication, transcription, translation, and posttranslational modifications. However, purified Ssr4 samples showed no DNA-binding activity, implying that the role of Ssr4 in genome-wide gene regulation could rely upon its acting as a cosubunit of the two complexes. These findings provide the first insight into an essential role of Ssr4 in the asexual cycle *in vitro* and *in vivo* of B. bassiana and highlights its importance for the filamentous fungal lifestyle.

**IMPORTANCE** Ssr4 is known to serve as a cosubunit of chromatin-remodeling SWI/SNF and RSC complexes in yeasts but has not been functionally characterized in fungi. This study unveils for the first time the pleiotropic effects caused by deletion of *ssr4* and its role in mediating global gene expression in a fungal insect pathogen. Our findings confirm an essential role of Ssr4 in hydrophobin biosynthesis and assembly required for growth, differentiation, and development of aerial hyphae for conidiation and conidial adhesion to insect surface and its essentiality for insect pathogenicity and virulence-related cellular events. Importantly, Ssr4 can regulate nearly one-fourth of all genes in the fungal genome in direct and indirect manners, including dozens involved in gene activity and hundreds involved in metabolism and/or transport of carbohydrates, amino acids, lipids, and/or inorganic ions. These findings highlight a significance of Ssr4 for filamentous fungal lifestyle.

## Abstract (precis)

Ssr4 is known to serve as a cosubunit of chromatin-remodeling SWI/SNF and RSC complexes in yeasts but has not been functionally characterized in fungi. This study unveils for the first time the pleiotropic effects caused by deletion of *ssr4* and its role in mediating global gene expression in a fungal insect pathogen. Our findings confirm an essential role of Ssr4 in hydrophobin biosynthesis and assembly required for growth, differentiation, and development of aerial hyphae for conidiation and conidial adhesion to insect surface and its essentiality for insect pathogenicity and virulence-related cellular events. Importantly, Ssr4 can regulate nearly one-fourth of all genes in the fungal genome in direct and indirect manners, including dozens involved in gene activity and hundreds involved in metabolism and/or transport of carbohydrates, amino acids, lipids, and/or inorganic ions. These findings highlight a significance of Ssr4 for filamentous fungal lifestyle.

## INTRODUCTION

Multisubunit SWI/SNF (switch/sucrose nonfermentable) and RSC (remodel the structure of chromatin) complexes can alter DNA-histone contacts in nucleosomes in an ATP-dependent manner and activate gene transcription and other chromatin-relating processes in model yeasts (1, 2). In Saccharomyces cerevisiae, the SWI/SNF complex serves as a chromatin-remodeling complex by binding to the binding sites of transcription factors, and its binding to both DNA and nucleosomes presents high affinity but little specificity (3, 4). Upon binding, the complex is recruited to specific genes for transcriptional activation through chromatin remodeling (5) and catalyzes formation of nucleosome dimers with mono- and dinucleosome templates assembled from histones and DNA of high-affinity nucleosome positioning sequence (6). The yeast RSC complex is required for cell viability (7, 8), mediates gene transcription by RNA polymerases (9–13), and plays a crucial role in cell cycle (14), kinetochore function (15), sister chromatid cohesion (16), and DNA repair (17, 18). These studies demonstrate important roles of both SWI/SNF and RSC complexes in most chromatin-related processes of S. cerevisiae.

A comprehensive study has unveiled that Schizosaccharomyces pombe SWI/SNF and RSC complexes consist of 12 and 13 components essential or nonessential for cell viability and are compositionally distinct from the S. cerevisiae counterparts comprising 12 and 17 components (1). The two complexes of S. pombe share four SSR (SWI/SNF and RSC) components (Ssr1 to Ssr4), which are all required for yeast viability. Also unveiled in the study, Ssr1/2 and Ssr3 are orthologous to Swi3 and Snf12, which act as nonessential SWI/SNF components, and to Rsc8 and Rsc6, which are essential RSC components in S. cerevisiae. In Candida albicans, some SWI/SNF or RSC components have also been characterized, such as Swi1 essential for formation of infective hyphae (19) and Snf6 required for differentiation of invasive hyphae in response to stress cues and growth on fermentable and nonfermentable carbon sources (20).

Despite intensive studies in model yeasts, either SWI/SNF or RSC components remain unexplored in filamentous fungal pathogens, leaving it unknown how they function in host-pathogen interactions. Revealed in our previous transcriptomic analysis, an orthologous Ssr4-coding gene was increasingly upregulated in the first 48-h infection course of Beauveria bassiana against the larvae of Plutella xylostella, a cosmopolitan lepidopteran pest (21). The transcript level of *ssr4* in the fungus-insect interaction increased 109-fold at 36 h versus 24 h postinfection and 2-fold at 48 h versus 36 h postinfection, while so striking transcript changes were not found in the coding genes of other SWI/SNF or RSC components (21). This implies that Ssr4 may serve as a critical player in the fungus-insect interaction. As a classic insect mycopathogen, B. bassiana has been widely applied for arthropod pest control. Its biological control potential depends mainly on an ability to infect the host through the normal route of cuticular penetration for entry into host hemocoel (22), where the penetrating hyphae turn into unicellular blastospores (also called hyphal bodies) to proliferate rapidly by yeast-like budding until host mummification to death (23–27), as well as an ability to overcome stress cues from host immunity defense and environment (28, 29). Our fungal genome survey revealed a wide existence of orthologous Ssr4 in filamentous fungi. This study seeks to elucidate a role of Ssr4 in the *in vitro* and *in vivo* asexual cycles of B. bassiana through multiphenotypic analyses of *ssr4* deletion/rescue mutants and transcriptomic analysis of differentially expressed genes (DEGs) in the Δ*ssr4* mutant versus a wild-type strain. In contrast to an essentiality of *ssr4* for the viability of S. pombe (1), our viable Δ*ssr4* mutant offers the first opportunity to gain in-depth insight into the regulatory role of Ssr4 in a fungus. As presented below, Ssr4 localizes in the nucleus and plays an essential role in the *in vitro* and *in vivo* asexual cycles of B. bassiana. Importantly, the expression of 2,517 genes, namely, one-fourth of the fungal genome, was significantly deregulated in the null mutant of *ssr4*.

## RESULTS

### Protein domain analysis, transcript levels, and subcellular localization of Ssr4 in B. bassiana.

**FIG 1:**
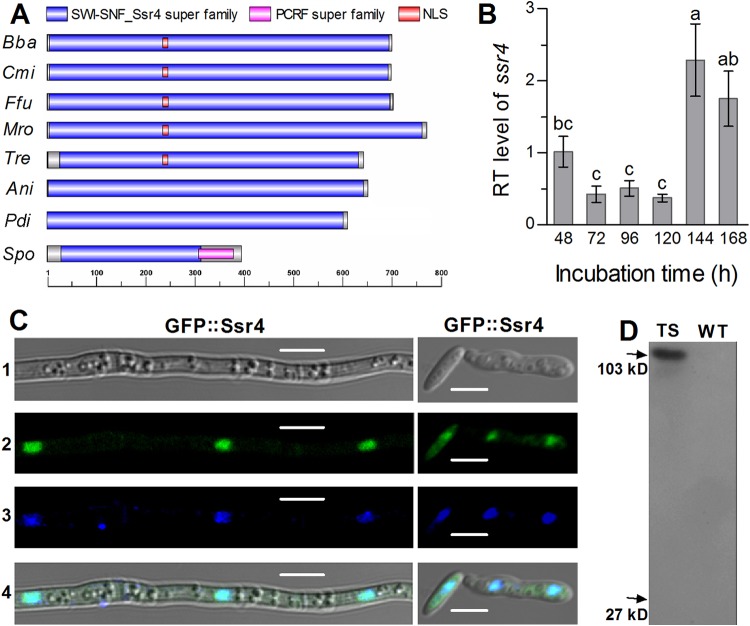
Properties of Ssr4 in B. bassiana. (A) Structural comparison of Ssr4 orthologs found in B. bassiana (Bba), Cordyceps militaris (Cmi), Fusarium fugikuroi (Ffu), Metarhizium roberstii (Mro), Trichoderma reesei (Tre), Aspergillus nidulans (Ani), Penicillium digitatum (Pdi), and S. pombe (Spo). The main domains and nuclear localization signal (NLS) were predicted at http://smart.embl-heidelberg.de and http://nls-mapper.iab.keio.ac.jp/cgi-bin/NLS_Mapper_form.cgi, respectively. (B) Relative transcript (RT) level of *ssr4* in the SDAY culture of a WT strain during a 7-day incubation at the optimal regimen with respect to a standard at the end of 48 h. Values are means ± standard deviations (SD) (error bars) from three cDNA samples. Different lowercase letters denote significant differences (Tukey’s HSD test, *P* < 0.05). (C) LSCM images for subcellular localization of GFP::Ssr4 fusion protein in the hyphal cells collected from 3-day-old SDBY culture and stained with DAPI. Panels 1 to 4 show bright, expressed, stained, and merged views of the same field, respectively. Note that the expressed signal (green) overlapped well with the stained signal (blue) in nuclei. Bars, 5 μm. (D) Western blots for GFP (27 kDa) and GFP::Ssr4 (102.9 kDa) in the protein extracts isolated from the 3-day-old SDBY cultures of transgenic strain (TS) versus WT (control) and probed with anti-GFP antibodies.

The nucleotide sequence of *ssr4* (locus tag BBA_01960) in the B. bassiana genome (30) is 2,231 bp long with two introns and encodes 701 amino acids (NCBI:protein accession no. or code EJP68925) with a molecular mass of 75.9 kDa and an isoelectric point of 4.74. The deduced protein sequence is more identical to the counterparts of filamentous fungi (34 to 84%) than to the S. pombe ortholog (26%) (see Fig. S1 in the supplemental material). In B. bassiana, Ssr4 features a large conserved SWI-SNF_Ssr4 domain (residues 6 to 695) and a nuclear localization signal (NLS) motif (residues 234 to 244) (Fig. 1A). Both the conserved domain and the NLS motif are also present in the orthologs of all examined filamentous fungi except Aspergillus nidulans and Penicillium digitatum. The latter two Eurotimyete species lack a predictable NLS motif. All of these orthologs are somewhat distinct from the S. pombe Ssr4, which has a peptide chain release factor (PCRF) domain overlapping the C-terminal end of the conserved domain and also lacks an NLS motif. Aside from Ssr4, most orthologs of the SWI/SNF or RSC components characterized in S. pombe (1) also exist in B. bassiana and four other filamentous fungi examined (see Table S1 in the supplemental material). Exceptionally, these filamentous fungi have one protein orthologous to both Ssr1 and Ssr2 and lack an ortholog of Snf30 in S. pombe, suggesting that only one of these components participates in filamentous fungal SWI/SNF complex.

10.1128/mSystems.00677-19.1 FIG S1 Phylogenetic analysis of Ssr4 orthologs found in B. bassiana and other fungi. Each fungal name was followed by the NCBI accession code of each protein and its sequence identity (as a percentage) to B. bassiana Ssr4 in parentheses. The bootstrap values of 1,000 replications are given at the nodes. The scale bar shows the branch length proportional to genetic distance assessed with the neighbor-joining method in MEGA7 at http://www.megasoftware.net. Download FIG S1, JPG file, 0.2 MB. Copyright © 2020 Shao et al. 2020 Shao et al. This content is distributed under the terms of the Creative Commons Attribution 4.0 International license.

10.1128/mSystems.00677-19.4 TABLE S1 A list of SWI/SNF and RSC components in yeasts and some filamentous fungi. Download Table S1, PDF file, 0.4 MB. Copyright © 2020 Shao et al. 2020 Shao et al. This content is distributed under the terms of the Creative Commons Attribution 4.0 International license.

The gene *ssr4* was consistently transcribed in the wild-type strain B. bassiana ARSEF 2860 (designated the wild type [WT]) during a 7-day incubation on Sabouraud dextrose agar plus yeast extract (SDAY) at the optimal regimen of 25°C in a light/dark (L/D) cycle of 12 h/12 h (Fig. 1B). Its transcription was significantly downregulated during days 3 to 5 and upregulated to a peak on day 6 with respect to the standard level at the end of a 2-day incubation.

The presence of an NLS motif in the B. bassiana Ssr4 implicates its possible localization in the nucleus. This was verified by expression of green fluorescence protein (GFP)-tagged Ssr4 fusion protein in the WT strain. As shown in laser scanning confocal microscopic (LSCM) images, the expressed fusion protein aggregated mainly in the nuclei of 4′,6′-diamidine-2′-phenylindole dihydrochloride (DAPI)-stained hyphal cells and merged well with the stained color (shown in blue) in the nuclei (Fig. 1C). However, the signal of the fusion protein in the cytoplasm was very weak. In a Western blot experiment to detect GFP alone and GFP::Ssr4 with anti-GFP antibody, only the fusion protein (92.9 kDa) was well detected in the protein extract isolated from the hyphal cells (Fig. 1D), indicating that the fusion protein was expressed as expected. The main localization of Ssr4 in the nuclei implies its involvement in nuclear events of *B.* bassiana.

### Ssr4 is required for aerial conidiation and cell hydrophobicity.

**FIG 2:**
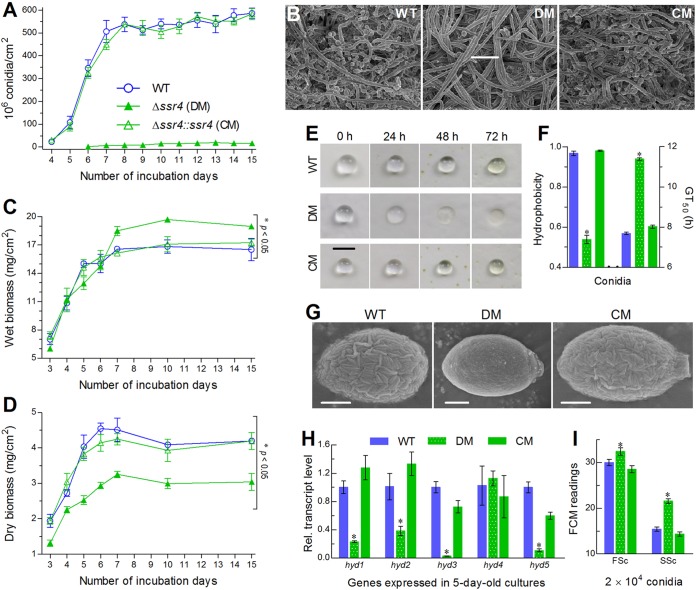
Ssr4 is required for aerial conidiation and cell hydrophobicity of B. bassiana. (A) Conidial yields quantified from the SDAY cultures during a 15-day incubation at the optimal regimen. The WT, deletion mutant (DM), and complemented mutant (CM) strains are shown. (B) SEM images for the status of conidiation in the 5-day-old SDAY cultures. Bar, 10 μm. (C and D) Wet (fresh) and dry biomass levels from the SDAY cultures, respectively. (E) Visual images for the stability of dew-like water droplets on the surfaces of the SDAY cultures over 72 h. Note that the water droplet gradually sank and disappeared in the Δ*ssr4* culture. Bar, 3 mm. (F) Conidial hydrophobicity assessed in an aqueous-organic system and median germination time (GT_50_) at 25°C. (G) SEM images for ultrastructural features of conidial coat. Bars, 10 μm. (H) Relative transcript levels of five hydrophobin genes (*hyd1* to *hyd5*) in the 5-day-old SDAY cultures of *ssr4* mutants versus WT. (I) Conidial size and complexity (density) indicated by the forward scatter (FSc) and side scatter (SSc) readings from flow cytometry (FCM) of 2 × 10^4^ conidia per sample. All SDAY cultures were initiated by spreading 100 μl of a 10^7^ conidia/ml suspension per plate (9-cm diameter). Values are means ± SD (error bars) from three replicates. Values that are significantly different (*P* < 0.05) for the Δ*ssr4* mutant and its control strains by Tukey’s HSD test are indicated by an asterisk.

The gene *ssr4* was deleted from the WT strain and rescued in an identified Δ*ssr4* mutant as described in Materials and Methods. The expected recombinant events were confirmed though PCR and Southern blotting analyses (Fig. S2) with paired primers and amplified probe (Table S2). As a consequence, the Δ*ssr4* mutant suffered extremely severe defect in aerial conidiation during a 15-day incubation at the optimal regimen on SDAY plates, which were spread with 100-μl aliquots of a 10^7^ conidia/ml suspension for culture initiation. The control (WT and complemented) strains produced a mean yield of 2.6 × 10^7^ conidia/cm^2^ on day 4 and approached a peak of ∼5.5 × 10^8^ conidia/cm^2^ on day 8 (Fig. 2A). Compared to the control strains, the mutant showed a 2-day delay in conidiation and a drastic (98%) decrease in final conidial yield during a 15-day incubation. Revealed by scanning electron microscopy (SEM), plenty of conidia formed in the 5-day-old cultures of the control strains, contrasting to a scarcity of conidia in the Δ*ssr4* culture comprising abundant hyphae not differentiated yet (Fig. 2B). We also measured biomass levels of each strain during the period and found that fresh (wet) biomass level increased significantly by 12 to 17% (Tukey’s honestly significance difference [HSD] test, *P* < 0.05) in the Δ*ssr4* cultures grown for 7 to 15 days in comparison to those measured from the cultures of the control strains (Fig. 2C). Intriguingly, dry biomass levels quantified from the 3- to 15-day-old Δ*ssr4* cultures were significantly (22 to 35%) lower than those of the control strains (Fig. 2D). Such measurements implicate a marked increase in hyphal hydrophility or water content of Δ*ssr4*. This implication was confirmed by dipping 10-μl aliquots of sterile water containing 0.02% Tween 80 on the surfaces of 4-day-old SDAY cultures and monitoring the 72-h stability of dew-like water droplets. As a consequence, the water droplets remained consistently intact on the culture surfaces of the control strains but gradually sank into the Δ*ssr4* culture and disappeared at the end of 72 h (Fig. 2E). These data indicate that Ssr4 is required for aerial conidiation but nonessential for biomass accumulation of *B.* bassiana on the SDAY plates spread evenly with conidia for culture initiation. The increased water content in hyphal cells and the water droplets disappeared on the culture surface of Δ*ssr4* implicate higher hydrophility of its hyphae and hence an important role for Ssr4 in sustaining hyphal hydrophobicity and aerial growth, which are critical for normal differentiation of aerial hyphae for conidiation (31).

10.1128/mSystems.00677-19.2 FIG S2 Generation and identification of B. bassiana *ssr4* mutants. (A) Diagram for the strategy of *ssr4* deletion. (B) The *ssr4* mutants identified via PCR (lanes 1 to 3) and Southern blotting (lanes 4 to 6) analyses with paired primers and amplified probe (Table S2). The wild-type (lanes 1 and 4), Δ*ssr4* (lanes 2 and 5), and Δ*ssr4*::*ssr4* (lanes 3 and 6) strains are shown. The detected PCR bands denote a fragment of 822 bp for the wild-type strain, a bar-inclusive fragment of 1,558 bp for Δ*ssr4* strain, and both fragments for Δ*ssr4*::*ssr4* strain, indicating that *ssr4* was disrupted by the deletion of a 224-bp fragment comprising partial promoter and coding sequences. Genomic DNAs were digested with HindIII at the marked sites for detection of *ssr4* via Southern blot hybridization with a probe of 461 bp, which enables detection of 1.6- and.2.3-kb fragments from the wild-type and Δ*ssr4* strains, respectively, and of both fragments from the Δ*ssr4*::*ssr4* strain. The difference (0.7 kb) of the two detected bands resulted from substitution of the deleted fragment by the *bar* marker (960 bp). Download FIG S2, JPG file, 0.2 MB. Copyright © 2020 Shao et al. 2020 Shao et al. This content is distributed under the terms of the Creative Commons Attribution 4.0 International license.

10.1128/mSystems.00677-19.5 TABLE S2 Paired primers used for targeted gene manipulation in B. bassiana. Download Table S2, PDF file, 0.3 MB. Copyright © 2020 Shao et al. 2020 Shao et al. This content is distributed under the terms of the Creative Commons Attribution 4.0 International license.

Aside from the severe conidiation defect, the *ssr4* deletion resulted in impaired conidial quality indicated by several features. The mutant conidia were compromised not only in viability due to a 3.7-h-longer time required for 50% germination (GT_50_) at optimal 25°C but also in hydrophobicity due to a 45% reduction in comparison to those of the control strains (Fig. 2F). Shown in the SEM images of conidia (Fig. 2G), the control strains had a well-defined rodlet layer of conidial coat comprising hydrophobin-borne fascicles or bundles (32). In contrast, such fascicles or bundles became obscure on the surfaces of apparently larger Δ*ssr4* conidia. Obviously, the Δ*ssr4* conidia were coated with distorted or fewer hydrophobin-borne bundles, coinciding well with the marked decrease in hydrophobicity. This observation is also supported by transcript levels of five hydrophobin family genes, which were repressed by 62 to 97% in the 5-day-old SDAY cultures of the Δ*ssr4* mutant versus the WT strain except for unaffected expression of *hyd4* (Fig. 2H). In addition, conidial size and complexity (density) were significantly increased in the absence of *ssr4* (Fig. 2I). The measured mean size of the mutant conidia coincided with the conidial size shown in the SEM image. All of these changes were well restored by targeted gene complementation and hence indicated an essential role of Ssr4 in maintenance of conidial quality, transcription and translation of hydrophobin genes, and assembly of hydrophobins into the bundles.

### Ssr4 is indispensable for fungal infection and virulence.

**FIG 3:**
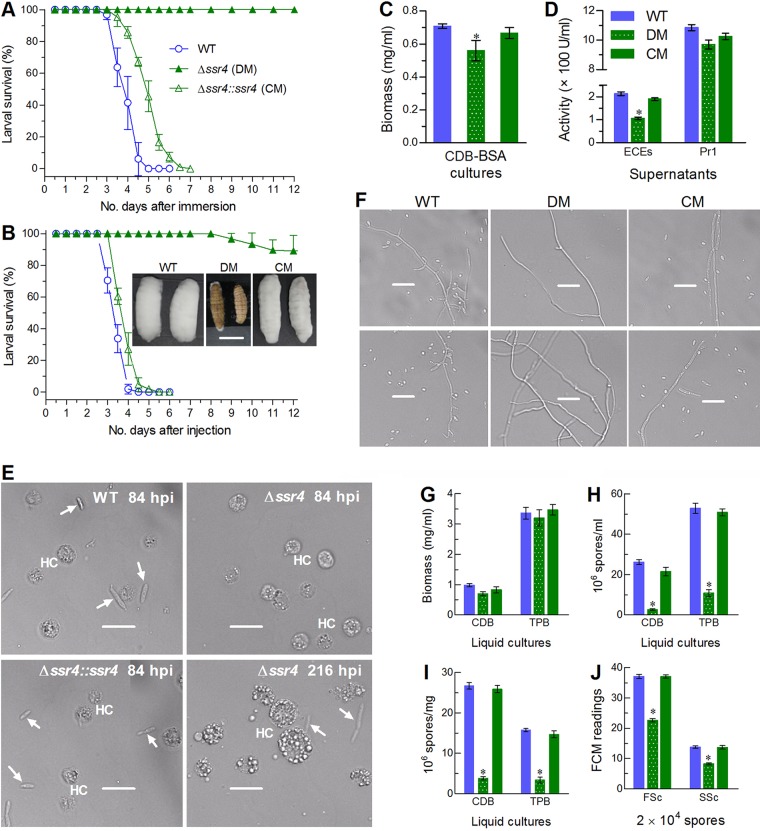
Ssr4 is indispensable for host infection and virulence of B. bassiana. (A and B) Survival trends of G. mellonella larvae after topical application (immersion) of a 10^7^ conidia/ml suspension for normal cuticle infection and intrahemocoel injection of ∼500 conidia per larva for cuticle-bypassing infection, respectively. The insets in panel B show fungal outgrowths on the surfaces of cadavers 5 days after death from injection. Bar, 10 mm. (C and D) Hyphal biomass levels and total activities of extracellular enzymes (ECEs) and Pr1 proteases quantified from the 3-day-old CDB-BSA cultures initiated with 10^6^ conidia/ml. (E) Microscopic images showing the presence or absence of hyphal bodies (indicated by white arrows) in the hemolymph samples taken from surviving larvae after injection. hpi, hours postinfection; HC, host hemocytes. Bars, 20 μm. (F to I) Microscopic images (scale: 20 μm), biomass levels, blastospore concentrations, and dimorphic transition rates quantified from 3-day-old CDB and TPB cultures, respectively. (J) Blastospore size and complexity (density) indicated by the FSc and SSc readings from flow cytometry (FCM) of 2 × 10^4^ blastospores per sample. *, *P* < 0.05 (Tukey’s HSD) for marked differences between Δ*ssr4* and its control strains. Error bars show SD from three replicates.

**FIG 4:**
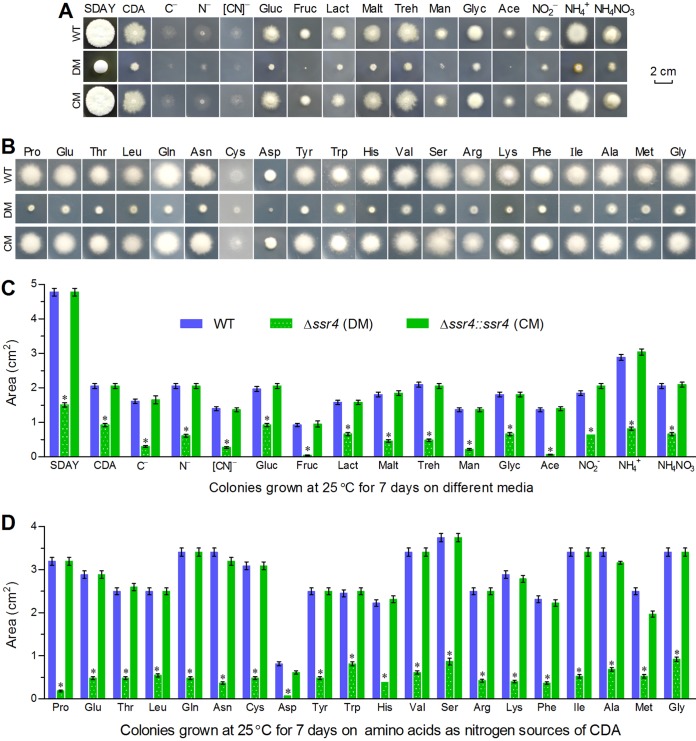
Essential role of Ssr4 in radial growth of B. bassiana. (A to D) Images and areas of fungal colonies initiated by spotting 1-μl aliquots of a suspension of 10^6^ conidia/ml and incubated 7 days at 25°C on the plates of rich SDAY, minimal CDA, and modified CDAs, which contained different carbon sources (Gluc, glucose; Fruc, fructose; Lact, lactose; Malt, maltose; Treh, trehalose; Glyc, glycerol; Ace, acetate) (A) or inorganic/organic (amino acids) nitrogen sources (B) or were free of carbon (C^–^), nitrogen (N^–^), or both ([CN]^–^). *, *P* < 0.05 (Tukey’s HSD) for marked differences between Δ*ssr4* and its control strains. Error bars show SD from three replicates.

The virulence of each strain against the fifth-instar larvae of greater wax moth (Galleria mellonella) was assayed by topical application (immersion) of a 10^7^ conidia/ml suspension for normal infection through cuticular penetration and intrahemocoel injection of ∼500 conidia (5 μl of a 10^5^ conidia/ml suspension) per larva for cuticle-bypassing infection. All treated larvae died from mummification by the control strains within 5 or 6 days postimmersion (Fig. 3A) and 4 or 5 days postinjection (Fig. 3B). In contrast, the Δ*ssr4* mutant caused no substantial mortality through the normal infection and only 11% mortality by the end of 12 days postinjection.

Noticeably, the deletion mutant was unable to grow out of cadavers, which were heavily covered with outgrowths of the control strains 5 days after death from the injection (inset images in Fig. 3B). This implied that the Δ*ssr4* cells inside the cadaver could not penetrate the cuticle for outgrowth. To verify this speculation, biomass levels and total activities of extracellular (proteolytic, chitinolytic, and lipolytic) enzymes (ECEs) and Pr1 proteases involved in cuticle degradation (22, 23, 27) were quantified from the 3-day-old submerged cultures of a 10^6^ conidia/ml suspension in Czapek-Dox broth (CDB), which contained the sole nitrogen source of 0.3% bovine serum albumin (BSA) for enzyme induction. As a result, the Δ*ssr4* mutant exhibited a 21% decrease in biomass level (Fig. 3C) and 50% and 11% reductions in total ECE and Pr1 activities (Fig. 3D) in comparison to the corresponding WT quantities. These data hint at a main link of reduced ESC and Pr1 activities to decreased biomass level in the submerged Δ*ssr4* culture and some other more important cellular events that might be involved in the mutant inability to infect insects through cuticular penetration.

For insight into drastic attenuation of the mutant virulence via the cuticle-bypassing infection, we examined the presence/absence and abundance of hyphal bodies in the hemolymph samples taken from the larvae surviving the injection. Such hyphal bodies are usually produced from penetrating hyphae after entry into host hemocoel and can propagate rapidly by yeast-like budding for acceleration of host mummification to death (24–27). The control strains formed abundant hyphal bodies (i.e., blastospores) in the samples at the time of 84 h postinjection, whereas the Δ*ssr4* mutant formed no hyphal body until 216 h postinjection (Fig. 3E). Blocking the dimorphic (hypha-blastospore) transition *in vivo* critical for fungal virulence was further examined in the following *in vitro* experiments, in which conidia were incubated 3 days in CDB and trehalose-peptone broth (TPB) mimicking host hemolymph. As a result, many more blastospores formed in the submerged cultures of the control strains than those of the Δ*ssr4* mutant (Fig. 3F) although their biomass levels in CDB or TPB were similar or close to each other (Fig. 3G). Intriguingly, blastospore concentrations in the CDB and TPB cultures of the Δ*ssr4* mutant decreased by 90% and 79%, respectively, in comparison to those estimated from the WT cultures (Fig. 3H). Thus, the dimorphic transition rate of the Δ*ssr4* mutant on the basis of its biomass level was reduced by 86% in CDB and 79% in TPB (Fig. 3I), accompanied by ∼40% decrease in both size and complexity of the blastospores produced in TPB (Fig. 3J). These results indicated a block of dimorphic transition *in vitro* or *in vivo* in the absence of *ssr4*.

Since the limited decreases of the ECE and Pr1 activities hardly accounted for the abolished host infection of the Δ*ssr4* mutant via cuticular penetration, we evaluated radial growth rates of each strain on rich SDAY, minimal CDA (CDB plus agar) and 34 CDAs amended with different carbon (sugars or polyols) or nitrogen (inorganic or organic) sources by spotting 1 μl of a 10^6^ conidia/ml suspension per plate for colony initiation. After a 7-day incubation at 25°C, the deletion mutant showed severe or extremely severe growth defects on all tested media. As illustrated in Fig. 4, the mean size of the Δ*ssr4* colonies decreased by 69% on SDAY, 55% on CDA, and 54 to 95% on all amended CDAs in comparison to the corresponding colony sizes of the control strains. Growth of the mutant was suppressed more on the carbon sources of fructose and sodium acetate (95%) than on sucrose and glucose (∼55%) and also on the nitrogen sources of most amino acids (77 to 94%) than on inorganic nitrogen sources tested (55 to 72%). These data demonstrate that Ssr4 is an essential sustainer of radial growth on nutrient-rich or scant substrata and hint at a main cause to interpret a complete loss of insect pathogenicity by the Δ*ssr4* mutant, which could be greatly suppressed in hyphal growth on oligotrophic insect integument as shown on the scant media and hence blocked in hyphal invasion into host.

### Ssr4 enables regulation of global gene expression.

**FIG 5:**
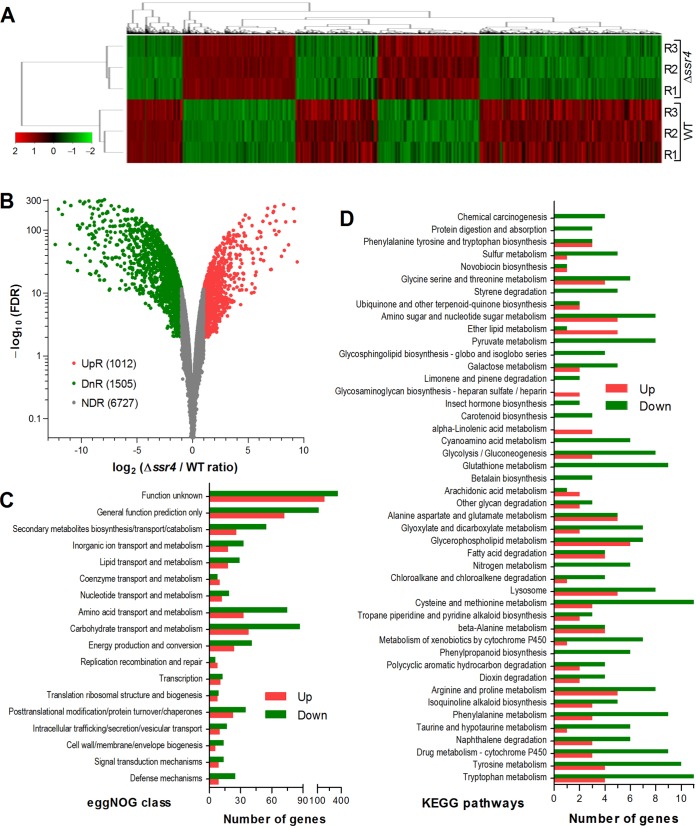
Genome-wide regulatory role of Ssr4 in B. bassiana. (A) Cluster analysis of differentially expressed genes (DEGs) in the transcriptomes generated from three 4-day-old SDAY cultures (three replicate cultures [R1, R2, and R3]) of Δ*ssr4* and WT strains grown at the optimal regimen of 25°C and 12-h/12-h L/D cycle. (B) Distributions of log_2_ ratios and FDR values for all genes significantly upregulated (UpR) (log_2_ ratio ≥ 1), downregulated (DnR) (log_2_ ratio ≤ −1), or not differentially regulated (NDR) (−1 < log_2_ ratio < 1). (C and D) Counts of DEGs significantly enriched in eggNOG function classes (C) and KEGG pathways (D).

Transcriptomes based on three 4-day-old SDAY cultures (replicates) of Δ*ssr4* and WT strains (Fig. 5A) were constructed and analyzed for in-depth insight into the essential role of Ssr4 in the *in vitro* and *in vivo* asexual cycles of B. bassiana. Among 9,308 genes mapped to the B. bassiana genome, 2,517 were differentially expressed in the Δ*ssr4* mutant versus WT strain at significant levels (Fig. 5B and Table S3), including 1,505 downregulated genes (–12.35 ≤ log_2_ ratio [*R*] ≤ –1.00) and 1,012 upregulated genes (1.00 ≤ log_2_ *R* ≤ 9.41), and 433 recognized as new genes. All DEGs accounted for 24.3% of the genome of B. bassiana (30). Such a high proportion of DEGs in the absence of *ssr4* indicates a requirement of Ssr4 for global gene activity. However, the developmental activator genes *brlA* (BBA_07544), *abaA* (BBA_00300), *wetA* (BBA_06126), and *vosA* (BBA_01023), which are evidently required for conidiation and conidial maturation of B. bassiana (33, 34), were markedly upregulated (log_2_ *R*, 6.67 for *brlA*, 3.38 for *abaA*, and 1.62 for *wetA*) or unaffected (*vosA*). None of the genes encoding signal transducers of the upstream developmental activation pathway required for activation of the critical *brlA* in A. nidulans (35–37), namely, *flbABCDE* (BBA_02968, BBA_06988, BBA_03181, BBA_07259, and BBA_01716, respectively) was differentially expressed although the upstream *fluG* (BBA_04942) was sharply repressed (log_2_ *R* = –8.35). The transcript changes of these development-required genes provided little insight into the severe conidiation defect of Δ*ssr4* under normal culture conditions.

10.1128/mSystems.00677-19.6 TABLE S3 A list of genes differentially expressed in the Δ*ssr4* mutant versus wild-type strain of B. bassiana. Download Table S3, XLSX file, 0.4 MB. Copyright © 2020 Shao et al. 2020 Shao et al. This content is distributed under the terms of the Creative Commons Attribution 4.0 International license.

Nearly 60% DEGs (526 up- and 962 downregulated genes) were significantly enriched in 21 function classes by eggNOG function classification (Fig. 5B and Table S4), including one class containing a single DEG (data not shown) and two uncharacterized classes containing 191 (general function predicted only) and 554 (function unknown) DEGs, respectively. There were 14, 24, 17, and 58 DEGs involved in DNA replication, transcription, translation, and posttranslational modifications, respectively. Many more downregulated genes were enriched in the carbohydrate transport and metabolism class than those upregulated (87 versus 38). Many repressed genes encode important enzymes essential for utilization of carbon sources, such as ribose 5-phosphate isomerase (BBA_04969) acting in both the pentose phosphate pathway and the Calvin cycle (38), six hexose transporters (BBA_05766, BBA_03013, BBA_00397, BBA_09477, BBA8728, and BBA5674) critical for glucose uptake (39), and most of the glycosyl hydrolases (BBA_03946, BBA_05847, BBA_09379, and BBA_08796) involved in the hydrolysis of glycosidic bonds, the processing of N-linked glycoproteins and the degradation of carbohydrates (40, 41). Twenty-seven other repressed genes encode membrane transporter proteins (major facilitator superfamily [MFS]), including two sugar transporters (BBA_09393 and BBA_06639). These genes are known to function in the movement of small solutes across cell membranes (42, 43) or act as sugar transporters (40, 44, 45). Up to 75 repressed genes were involved in amino acid transport and metabolism, contrasting to only 33 upregulated in the class. The repressed genes encode five aminotransferases (BBA_08426, BBA_09446, BBA_03201, BBA_06530, and BBA_04988) involved in transamination reaction and the synthesis and breakdown of amino acids (41), five amino acid permeases essential for the transport of amino acids into cells (44, 46), five carboxypeptidases (BBA_02206, BBA_02865, BBA_00850, BBA_05763, and newGene_144) involved in the hydrolysis of peptide bond, the mediation of translocation across the endoplasmic reticulum and the targeting of molecules to the vacuole (47), three γ-glutamyl transpeptidases (BBA_05887, BBA_06332, and BBA_04223) involved in the γ-glutamyl cycle as a pathway of glutathione synthesis and degradation (48), and three dihydrodipicolinate synthases (BBA_06627, BBA_02585, and BBA_05664) essential for lysine biosynthesis (49). Also, more repressed genes than those upregulated were functionally enriched in energy production and/or conversion (41 versus 24), lipid transport and metabolism (29 versus 18), inorganic ion transport and metabolism (33 versus 18), and secondary metabolite biosynthesis, transport and catabolism (55 versus 26) classes. Apparently, differential expression and malfunction of so many important genes associated with nutritional utilization and energy production/conversion were causative of not only the severe growth defects of the Δ*ssr4* mutant on all tested carbon and nitrogen sources but also both abolishment of its insect pathogenicity through cuticular penetration and great attenuation of its virulence through intrahemocoel injection.

10.1128/mSystems.00677-19.7 TABLE S4 Functional classification of differentially expressed genes in the Δ*ssr4* mutant versus wild-type strain of B. bassiana. Download Table S4, XLSX file, 0.2 MB. Copyright © 2020 Shao et al. 2020 Shao et al. This content is distributed under the terms of the Creative Commons Attribution 4.0 International license.

Aside from the eggNOG classification, 334 DEGs (236 down- versus 98 upregulated) were significantly (*q *< 0.05) enriched in 46 Kyoto Encyclopedia of Genes and Genomes (KEGG) pathways (Fig. 5D and Table S5). Many repressed genes were enriched in the metabolism pathways of carbohydrates (amino sugars and nucleotide sugars) and amino acids (tryptophan, tyrosine, phenylalanine, β-alanine, cysteine, methionine, arginine, and proline), providing further insights into the fungal phenotypes markedly impaired in the absence of *ssr4*.

10.1128/mSystems.00677-19.8 TABLE S5 KEGG pathways enriched with differentially expressed genes in the Δ*ssr4* mutant versus wild-type strain of B. bassiana. Download Table S5, XLSX file, 0.04 MB. Copyright © 2020 Shao et al. 2020 Shao et al. This content is distributed under the terms of the Creative Commons Attribution 4.0 International license.

### Ssr4 has no DNA-binding activity.

Electrophoretic mobility shift assay (EMSA) for detecting protein-nucleic acid interactions (50) was conducted to examine whether purified Ssr4 extract can bind to amplified promoter DNA fragments of six selected genes, which were sharply repressed (log_2_ ratio < –8) in the absence of *ssr4* (Fig. S3A). The resultant gels showed no sign of any DNA fragment bound by 0.8 to 4.0 μg of purified Ssr4 extract through agarose gel electrophoresis (Fig. S3B). The staining of each gel with Coomassie brilliant blue also revealed no activity of each protein sample bound to any DNA fragment. Obviously, Ssr4 had no DNA-binding activity, excluding a possibility of its acting as a transcription factor in B. bassiana.

10.1128/mSystems.00677-19.3 FIG S3 Electrophoretic mobility shift assay (EMSA) for the binding activity of Ssr4 to promoter DNA fragments in B. bassiana. (A) Paired primers used for amplifying the promoter DNA fragments of six genes from the genomic DNA of a wild-type strain (WT). Note that the selected genes (log_2_ *R* < −8) were drastically repressed in the Δ*ssr4* strain versus WT strain. (B) EMSAs for the binding activity of Ssr4 to each of six DNA fragments amplified. Target protein (Ssr4) samples were extracted from the cell lysate of Escherichia coli, in which the *ssr4* cDNA was expressed and purified through affinity chromatography column, dialysis, and concentration. Aliquots of 4 μl DNA extract were uploaded for reactions with 0.8, 1.6, 2.4, 3.2, and 4.0 μg (lanes 2 to 6) of purified protein extract through agarose gel electrophoresis (upper panel of each EMSA), respectively. For negative controls, lanes 1 and 7 were uploaded with only 4 μl DNA extract and only 4 μg protein extract, respectively. All gels were stained with Coomassie brilliant blue (lower panel of each EMSA) to show the binding activity of each protein sample to a given DNA fragment. Note that there is no sign of binding activity of purified Ssr4 to any of the examined promoter DNA fragments. Download FIG S3, JPG file, 1.1 MB. Copyright © 2020 Shao et al. 2020 Shao et al. This content is distributed under the terms of the Creative Commons Attribution 4.0 International license.

## DISCUSSION

As a cosubunit of SWI/SNF and RSC complexes, Ssr4 is required for yeast cell viability (1), leaving its functions unexplored yet. In this study, orthologous Ssr4 was proven to localize in the nucleus, act as a key player in genome-wide gene expression, and hence have pleiotropic effects on radial growth, cell hydrophobicity and differentiation, aerial conidiation, insect pathogenicity, and virulence-related cellular events in B. bassiana. These findings provide the first in-depth insight into an essential role for Ssr4 in the *in vitro* and *in vivo* asexual cycles of a filamentous fungal pathogen. Importantly, our transcriptomic analysis unveiled nearly one-fourth of all *B.* bassiana genes to be transcriptionally mediated by Ssr4 in direct and indirect manners, making it possible to understand its pleiotropic effects, as discussed below.

Asexual development is critical for the survival and dispersal of a filamentous fungus and genetically controlled by sequential activation of *brlA*, *abaA*, and *wetA* in the central developmental pathway and downstream of *vosA* (35–37). The four genes were proven to be required for aerial conidiation and conidial maturation of B. bassiana due to abolished conidiation in the absence of *brlA* or *abaA* (34) and greatly reduced (98% or 88%) conidial yield and impaired conidial quality in the absence of *wetA* or *vosA* (33). In this study, deletion of *ssr4* resulted in a 98% decrease in conidiation capacity, but none of the four genes was repressed at the transcriptional level, suggesting that some other factors were responsible for the severe conidiation defect. Aside from the developmental activators, multifunctional hydrophobins are well-known to take part in aerial hyphal growth and differentiation essential for conidiophore development and conidiation in filamentous fungi (31). In B. bassiana, conidial hydrophobicity was reported to be significantly lowered by the deletion of *hyd1* (class I hydrophobin gene) that resulted in bald conidial surface with altered rodlet fascicles/bundles or the deletion of *hyd2* (class II hydrophobin gene) that led to distorted conidial surface bundles with truncated/incomplete rodlets, and much more reduced by *hyd1-hyd2* double deletion (32). However, impacts of the single and double deletions on the fungal conidiation were not disclosed in the previous study. In this study, *hyd1*, *hyd2*, and two other hydrophobin-like genes (*hyd3* and *hyd5*) were markedly repressed in the absence of *ssr4*. In the Δ*ssr4* mutant, the reduced transcript levels of these genes correlated well with impaired hydrophobicity of hyphal culture, which featured an enhanced capability of water absorption and an increased water content. As a result of this correlation, hyphal differentiation and aerial conidiation were severely blocked, as shown in the SEM images. The limited conidia produced by the Δ*ssr4* mutant were also featured by an impaired coat with obscure, distorted, and fewer hydrophobin rodlet bundles and hence a marked decrease in hydrophobicity. Taken together, a block of aerial conidiation in the absence of *ssr4* is attributable to blocked differentiation of more hydrophilic hyphae, in which the *hyd* genes required for hydrophobin biosynthesis and assembly were largely downregulated at the transcription level, but not associated with any effect on the central pathway. In other words, Ssr4 mediates hyphal differentiation and aerial conidiation of B. bassiana by its involvement in transcription of the *hyd* family genes.

Our Δ*ssr4* mutant was unable to cause any insect mortality through the cuticle infection and also lost ∼90% of its lethal action against the model insect tested via cuticle-bypassing infection by intrahemocoel injection. This highlights the indispensability of Ssr4 for fungal pathogenicity and virulence and that it plays a role as a supervirulence factor in B. bassiana. Fungal lethal action against insects depends on not only an ability to infect the host by hyphal penetration through insect cuticle but also intrahemocoel transition of penetrating hyphae to unicellular blastospores for rapid proliferation by yeast-like budding until host death from mummification. In general, hyphal penetration through insect cuticle relies upon the cuticle-degrading activities of secreted enzymes, including Pr1 family proteases (22, 51). In B. bassiana, pathogenicity abolished or greatly reduced via cuticle infection is often correlated with hindered secretion of such enzymes in the absence of one of some genes that function in different pathways, such as those encoding the Hsp40 family member Mas5 (23), the Na^+^/H^+^ antiporter Nhx1 (24), the eisosome proteins Pil1A and Pil1B (25), the blue-light receptor VVD (27), and the histone acetyltransferase Gcn5 (26) or deacetylase Rpd3 (52). In this study, however, complete abolishment of cuticle infection by the *ssr4* deletion is unlikely an outcome from a limited decrease in total activity of secreted enzymes involved in cuticle degradation. Instead, this abolished phenotype is likely due to the severe Δ*ssr4* defects in conidial adhesion, germination, and hyphal growth. Impaired coat, lowered hydrophobicity, and increased size of the mutant conidia could have reduced conidial adhesion to the surface of the insect. This inference is supported by a correlation of reduced conidial adhesion with blocked cuticle infection in the double deletion mutant of *hyd1* and *hyd2* (32), both of which were downregulated with *hyd3* and *hyd5* in the Δ*ssr4* mutant. Aside from the reduced adhesion, the Δ*ssr4* mutant could be slower in conidial germination and greatly suppressed in hyphal growth on oligotrophic insect integument, as indicated by its delayed germination *in vitro* and severe growth defects on nutritionally limited media. Such growth defects of the Δ*ssr4* mutant are close to those caused by single-gene deletions of some other SWI/SNF subunits in C. albicans, such as an inability for the Δ*swi1* mutant to form true hyphae essential for pathogenicity (19) and hyphal growth defects of the Δ*snf6* mutant on fermentable and nonfermentable carbon sources (20). On the other hand, the fungal virulence via cuticle-bypassing infection is also largely attenuated by inactivated genes as mentioned above due to repressed expression of developmental activator genes associated with blocked transition of hyphae to blastospores and vice versa. Such dimorphic transition has proved a developmental process governed by the key developmental activator gene *brlA* or *abaA* in *B.* bassiana (34). In this study, injection of the Δ*ssr4* conidia resulted in a very low (only 11%) insect mortality, accompanied by a failure to form hyphal bodies *in vivo* until 216 h postinjection and a drastic reduction in dimorphic transition *in vitro*. The delayed formation of hyphal bodies after injection may account for the low insect mortality, but it is not linked to either accumulation of hyphal biomass in the submerged cultures or repressed expression of any developmental activator gene. Our transcriptomic analysis revealed many repressed genes involved in different pathways of carbon/nitrogen metabolism and transport. These repressed genes could exert comprehensive effects on cell growth, differentiation, and development, helping us to understand severe growth defects of the Δ*ssr4* mutant on various carbon and nitrogen sources, a complete loss of its pathogenicity via the cuticle infection, and a 90% loss of its virulence via cuticle-bypassing infection.

The pleiotropic effects and genome-wide regulatory role of Ssr4 help us to understand an increasingly upregulated expression of *ssr4* during the first 48-h period of B. bassiana infection comprising sequential steps of conidial germination, hyphal penetration through cuticle and hemocoel colonization (21). More than 24% of all genes regulated by Ssr4 is far beyond those regulated by Snf6 (∼10%) in C. albicans (20) and Reil1-like protein (13.5%), a pre-60S subunit export factor that was also increasingly upregulated at the transcriptional level in the 48-h infection course and involved in nutritional metabolism and transport required for the asexual cycle *in vitro* and *in vivo* of B. bassiana (53). Importantly, a large number of genes downregulated in the Δ*ssr4* mutant are involved in metabolism and/or transport of carbohydrates, amino acids, lipids and inorganic ions, energy production and conversion, and biosynthesis, transport, and catabolism of secondary metabolites. These genes positively regulated by Ssr4 could exert fundamental impacts on nutrition utilization, energy supply, and intracellular ion homeostasis. Intriguingly, our EMSA results excluded the possibility of Ssr4 acting alone as a transcription factor, hinting at the likelihood that the mediation of global gene expression by Ssr4 may rely on the role of its taking part in the chromatin remodeling of the SWI/SNF complex, which can bind to the binding sites of transcription factors in model yeast (3, 4). Notably, 113 genes differentially expressed in the Δ*ssr4* mutant are functionally involved in DNA replication, transcription, translation, and posttranslational modifications, thereby expanding the role of Ssr4 in genome-wide transcription regulation. Therefore, we infer that Ssr4 regulates global gene expression in B. bassiana by acting directly as a subunit of the chromatin-remodeling complex or indirectly through transcriptional regulation of those genes that enable mediation of gene activity.

Altogether, our findings uncover for the first time the pleiotropic effects of Ssr4 required for the asexual cycle *in vitro* and *in vivo* of B. bassiana and also its essential role in global gene expression and recall research attention to molecular mechanisms underlying the role of Ssr4 in the chromatin-remodeling SWI/SNF complex and interactions between Ssr4 and other subunits of the complex in filamentous fungal pathogens.

## MATERIALS AND METHODS

### Subcellular localization of Ssr4 in B. bassiana.

The coding sequence of *ssr4* revealed by previous analysis of transcriptomes (21) was amplified from the cDNA of the WT strain with paired primers (see Table S2 in the supplemental material). The protein sequence deduced from the coding sequence and those of Ssr4 orthologs found in the genomic databases of S. pombe and several filamentous fungi via online BLAST analysis (https://blast.ncbi.nlm.nih.gov/Blast.cgi) were aligned with the SMART program (http://smart.embl-heidelberg.de) for structural comparison, followed by analysis of their phylogenetic relationships with MEGA7 at http://www.megasoftware.net. A nuclear localization signal was predicted from each ortholog using cNLS Mapper software at https://omictools.com/cnls-mapper-tool.

The N terminus of the amplified *ssr4* cDNA was fused to the C terminus of *gfp* (GenBank accession no. or code U55763), inserted into linearized pAN52-bar, and transformed into the WT as described previously (53). A transgenic strain best expressing the fusion gene *gfp*::*ssr4* was chosen for incubation and full conidiation at 25°C on SDAY (4% glucose, 1% peptone, and 1.5% agar plus 1% yeast extract). The resultant conidia were suspended in SDBY (i.e., agar-free SDAY) and incubated 3 days on a shaking (150 rpm) bed at 25°C. Hyphal cells collected from the culture were stained 30 min with the nucleus-specific dye DAPI (4′,6′-diamidine-2′-phenylindole dihydrochloride; Sigma) at ambient temperature and visualized through LSCM for subcellular localization of expressed fusion protein. The protein extracts isolated from the 3-day-old cultures of the WT and transgenic strains were probed with mouse monoclonal anti-GFP antibodies (Cell Signaling Technology, Boston, MA, USA) for Western blots of GFP alone and GFP::Ssr4 to verify whether the fusion protein was expressed as expected.

### Generation of *ssr4* mutants.

The gene *ssr4* was deleted by homogenous recombination of its 5′ and 3′ fragments separated by *bar* maker (p0380-5′ssr4-bar-3′ssr4) in the WT and rescued in an identified deletion mutant by ectopic integration of a cassette consisting of its full-length sequence and *sur* marker (p0380-sur-ssr4) through *Agrobacterium*-mediated transformation, as described previously (53). Briefly, the deletion plasmid was constructed by amplifying the 5′ and 3′ coding/flanking fragments (1,260 and 1,381 bp, respectively) of *ssr4* from the genomic DNA of the WT strain with paired primers (Table S2) under the action of La*Taq* DNA polymerase from Promega (Madison, MI, USA) and inserting them into appropriate enzyme sites in our backbone plasmid p0380-bar. The complementing plasmid was constructed by amplifying a full-length coding/flanking sequence of *ssr4* (3,833 bp in total) from the WT DNA and inserting it into p0380-sur-gateway to take the place of the gateway fragment. After transformation, putative mutant colonies were screened by the *bar* resistance to phosphinothricin (200 μg/ml) or the *sur* resistance to chlorimuron ethyl (10 μg/ml), followed by PCR and Southern blot analyses with paired primers and amplified probe (Table S2) to verify the expected recombination events. Positive Δ*ssr4* and Δ*ssr4*::*ssr4* mutants were evaluated together with the WT strain in the following experiments of three independent cultures (replicates) or samples from the cultures.

### Experiments for pleiotropic effects.

The 1-μl aliquots of a 10^6^ conidia/ml suspension were centrally spotted on the plates of SDAY, CDA (3% sucrose, 0.3% NaNO_3_, 0.1% K_2_HPO_4_, 0.05% KCl, 0.05% MgSO_4_ and 0.001% FeSO_4_ plus 1.5% agar) and 34 CDA-derived media, which were prepared by deleting 3% sucrose or 0.3% NaNO_3_ or both from the standard CDA, replacing the carbon source with 3% glucose, fructose, lactose, maltose, trehalose, mannitol, glycerol, or acetate (sodium acetate [NaAc]), and replacing the nitrogen source with 0.3% NH_4_Cl, NaNO_2_, NH_4_NO_3_ or one of 20 amino acids, respectively. After a 7-day incubation at 25°C, the mean diameter of each colony was estimated with two measurements taken perpendicular to each other across the colony center to calculate colony area as a radial growth index of each strain on each medium.

Conidiation capacity was quantified from the SDAY cultures initiated by spreading 100 μl of a 10^7^ conidia/ml suspension per plate (9-cm diameter) and incubated 15 days at the optimal regimen of 25°C and 12-h/12-h light/dark (L/D) cycles. From day 4 onwards, three plugs (5-mm diameter) were taken daily from each plate culture using a cork borer. All conidia were released into 1 ml of 0.02% Tween 80 from each plug by supersonic vibration. The conidial concentration in the resultant suspension was assessed with a hemocytometer and converted to the number of conidia per unit area (squared centimeters) of plate culture. For assessment of biomass level, SDAY plates overlaid with cellophane were spread with 100-μl aliquots of the same conidial suspension for culture initiation, followed by a 15-day incubation at the same regimen. During the period of incubation, biomass levels were quantified from the fresh culture (wet biomass) of each plate and the same culture dried 2 h at 70°C (dry biomass), respectively. To reveal hydrophobic and hydrophilic features of hyphal cultures, 10-μl aliquots of sterile water containing 0.02% Tween 80 were dipped onto the surfaces of 4-day-old SDAY cultures initiated with the same method. The dew-like water droplets were monitored for their stability on the culture surfaces of different strains during a period of 72 h at 25°C. In addition, conidiation status of each strain in the 5-day-old SDAY cultures and ultrastructural changes on the surfaces of conidia were examined via scanning electron microscopy (SEM) after pretreatments as described elsewhere (27, 32).

Conidial virulence of each strain was assayed on G. mellonella larvae in two infection modes. Briefly, three batches of ∼35 larvae per batch were separately immersed for 10 s in 30 ml of a 10^7^ conidia/ml suspension for normal infection through cuticular penetration. Alternatively, 5 μl of a 10^5^ conidia/ml suspension was injected into the hemocoel of each larva in each batch for cuticle-bypassing infection. All batches treated with each strain in either infection mode were maintained at 25°C for 12 days and monitored every 12 or 24 h for survival/mortality records. During the period of monitoring, hemolymph samples were taken from surviving larvae and examined for the presence/absence and abundance of hyphal bodies under a microscope. Cadavers 5 days after death were observed to show an ability for intrahemocoel cells of each strain to penetrate insect cuticle for outgrowth and conidiation on cadaver surfaces.

Infection- and virulence-related cellular events of all strains tested were examined. First, conidial hydrophobicity as an important index of conidial adhesion to insect integument was assessed in a diphase (aqueous/organic) system as described previously (54, 55). Second, total extracellular enzyme (ECE) and Pr1 activities essential for cuticle degradation were quantified from the supernatants (crude extracts) of 3-day-old submerged cultures in CDB (i.e., agar-free CDA) by reading optical densities at 442 nm (OD_442_) and 410 nm (OD_410_), respectively, as described elsewhere (23, 26, 51). The submerged cultures were initiated with 50-ml aliquots of a 10^6^ conidia/ml suspension in CDB containing the sole nitrogen source of 0.3% BSA for enzyme induction. One unit of enzyme activity was defined as the enzyme amount required for an increase of each OD value by 0.01 after 1-h reaction of each extract versus a control; total activity was expressed as units per milliliter of supernatant with respect to dry biomass level estimated from the culture. Third, 20-ml aliquots of a 10^6^ conidia/ml suspension in CDB and TPB (amended CDB containing 3% trehalose [sole carbon source] and 0.5% peptone [sole nitrogen source]) were incubated by shaking (150 rpm) for 3 days at 25°C. Blastospore concentration and dry biomass level (in milligrams per milliliter) were quantified from each culture. The two quantities were used to compute dimorphic transition rate (number of blastospores per milligram of biomass) *in vitro*. Finally, standardized suspensions (10^7^ cells/ml) were prepared with three samples of conidia from the SDAY cultures or samples of blastospores from the TPB cultures of each strain. The size and complexity of conidia or blastospores were assessed with the respective readings from forward scatter (FSc) and side scatter (SSc) detectors in flow cytometry of 2 × 10^4^ conidia or blastospores per sample.

All data from the experiments of three replicates were subjected to one-way (strain) analysis of variance, followed by Tukey’s honestly significant difference (HSD) test for phenotypic changes among the tested fungal strains.

### Transcriptional profiling.

Aliquots of 100 μl of the 10^7^ conidia/ml suspension were spread onto cellophane-overlaid SDAY plates and incubated 5 or 7 days at the optimal regimen. Total RNAs were extracted daily from the WT cultures during the 7-day cultivation or from the 5-day-old cultures of the Δ*ssr4* mutant and control strains using an RNAiso Plus kit (TaKaRa, Dalian, China) and reverse transcribed into cDNAs using a PrimeScriptH^RT^ reagent kit (TaKaRa), respectively. Three samples of each cDNA were used as the templates to assess transcript levels of *ssr4* in the daily WT cultures and of five hydrophobin genes (*hyd1* to* hyd5*) in the 5-day-old cultures of the Δ*ssr4* mutant and control strains through real-time quantitative PCR (qPCR) with paired primers (Table S2) under the action of SYBR Premix Ex Taq (TaKaRa). The fungal 18S rRNA was used as an internal control. The threshold cycle (2^−ΔΔCT^) method was used to calculate the relative transcript level of *ssr4* over the incubation days of the WT strain with respect to a standard transcript at the end of 48-h incubation and of each *hyd* gene in the s*sr4* mutants with respect to the WT standard. Each qPCR was repeated three times using cDNA samples derived from independent cultures.

### Transcriptomic analysis.

Three 4-day-old cultures (replicates) of Δ*ssr4* and WT strains grown on cellophane-overlaid SDAY plates were prepared as mentioned above and sent to Microanaly Gene Co. (Shanghai, China) for transcriptome constructs and analysis. Extraction of total RNA from each culture, isolation of mRNA from the total RNA, fragmentation of mRNA, and syntheses of first- and second-strand cDNAs followed the same protocols of transcriptome construction as described previously (53). Each of the double-stranded cDNAs was purified and end repaired, followed by adding a single adenine to the ends of the cDNA molecules. The final cDNA library was generated by adding proper adaptors to the cDNA and sequenced on an Illumina Novaseq 6000 platform.

All clean tags generated through filtration of raw reads from the cDNA sequencing were mapped to the *B.* bassiana genome (30). All data were normalized as fragments per kilobase of exon per million fragments mapped (FPKM). Differentially expressed genes (DEGs) were identified in terms of log_2_ (Δ*ssr4*/WT ratio) ≤ −1 (downregulated) or ≥ 1 (upregulated) at the significance level of a false-discovery rate (FDR) of less than 0.01. All identified DEGs were annotated with known or putative gene information in the nonredundant NCBI protein databases and subjected to eggNOG function classification with eggNOG v4.5 (http://eggnog.embl.de). All function classes were enriched to four categories: cellular process and signaling, information storage and processing, metabolism, and uncharacterized. Kyoto Encyclopedia of Genes and Genomes (KEGG) analysis (http://www.genome.jp/kegg/) was also performed to enrich DEGs to various KEGG pathways at the significance level of *q *< 0.05.

### EMSA for binding activity of Ssr4 to promoter DNA fragments.

Electrophoretic mobility shift assay (EMSA) was conducted as described previously (53). Briefly, Ssr4 expressed in competent Escherichia coli cells was detected in cell lysates through sodium dodecyl sulfate-polyacrylamide gel electrophoresis (SDS-PAGE), followed by purification through affinity chromatography column, dialysis, and standardization to 0.4 mg/ml. The purified protein samples were used as the templates to detect the binding activity of Ssr4 to each of six promoter DNA fragments amplified from the genomic DNA of the WT with paired primers (Fig. S3A). The 4-μl aliquots of each DNA extract were mixed with 2 to 10 μl (0.8 to 4.0 μg) of the purified protein extract, respectively, in 10-μl aliquots of binding buffer comprising 25 mM HEPES (2-[4-(2-hydroxyethyl)-1-piperazinyl] ethanesulfonic acid [pH 7.4]), 50 mM KCl, 5 mM MgCl_2_, 0.5 mM EDTA, 1 mM dithiothreitol, and 5% glycerol. The combinations of 4 μl DNA extract with no protein extract and of 10 μl protein extract with no DNA extract were used as negative controls in each EMSA. All reactions lasted for 30 min at ambient temperature, followed by agarose gel electrophoresis for detection of Ssr4-bound DNA. The resultant gels were stained with Coomassie brilliant blue and rinsed repeatedly in washing buffer for visualization of the targeted protein.

### Data availability.

All data generated or analyzed during this study are included in the published paper and associated supplemental files. All transcriptomic data aside from those reported in supplemental files (Tables S3 to S5) of this paper are available at the NCBI’s Gene Expression Omnibus under the accession no. GSE132211.

## References

[1] Monahan BJ, Villén J, Marguerat S, Bähler J, Gygi SP, Winston F 2008 Fission yeast SWI/SNF and RSC complexes show compositional and functional differences from budding yeast. Nat Struct Mol Biol 15:873–880. doi:10.1038/nsmb.1452. 18622392 PMC2559950

[2] Whiteway M, Tebung WA, Choudhury BI, Rodríguez-Ortiz R 2015 Metabolic regulation in model ascomycetes−adjusting similar genomes to different lifestyles. Trends Genet 31:445 − 453. doi:10.1016/j.tig.2015.05.002. 26051071

[3] Hassan AH, Prochasson P, Neely KE, Galasinski SC, Chandy M, Carrozza MJ, Workman JL 2002 Function and selectivity of bromodomains in anchoring chromatin-modifying complexes to promoter nucleosomes. Cell 111:369–379. doi:10.1016/S0092-8674(02)01005-X. 12419247

[4] Martens JA, Winston F 2003 Recent advances in understanding chromatin remodeling by Swi/Snf complexes. Curr Opin Genet Dev 13:136–142. doi:10.1016/S0959-437X(03)00022-4. 12672490

[5] Neely KE, Hassan AH, Brown CE, Howe L, Workman JL 2002 Transcription activator interactions with multiple SWI/SNF subunits. Mol Cell Biol 22:1615–1625. doi:10.1128/mcb.22.6.1615-1625.2002. 11865042 PMC135607

[6] Krajewski WA, Vassiliev OL 2010 The *Saccharomyces cerevisiae* Swi/Snf complex can catalyze formation of dimeric nucleosome structures *in vitro*. Biochemistry 49:6531–6540. doi:10.1021/bi1006157. 20608642

[7] Laurent BC, Yang X, Carlson M 1992 An essential *Saccharomyces cerevisiae* gene homologous to SNF2 encodes a helicase-related protein in a new family. Mol Cell Biol 12:1893–1902. doi:10.1128/mcb.12.4.1893. 1549132 PMC369633

[8] Cairns BR, Lorch Y, Li Y, Zhang M, Lacomis L, Erdjument-Bromage H, Tempst P, Du J, Laurent B, Kornberg RD 1996 RSC, an essential, abundant chromatin-remodeling complex. Cell 87:1249–1260. doi:10.1016/s0092-8674(00)81820-6. 8980231

[9] Angus-Hill ML, Schlichter A, Roberts D, Erdjument-Bromage H, Tempst P, Cairns BR 2001 A Rsc3/Rsc30 zinc cluster dimer reveals novel roles for the chromatin remodeler RSC in gene expression and cell cycle control. Mol Cell 7:741–751. doi:10.1016/s1097-2765(01)00219-2. 11336698

[10] Damelin M, Simon I, Moy TI, Wilson B, Komili S, Tempst P, Roth FP, Young RA, Cairns BR, Silver PA 2002 The genome-wide localization of Rsc9, a component of the RSC chromatin-remodeling complex, changes in response to stress. Mol Cell 9:563–573. doi:10.1016/S1097-2765(02)00475-6. 11931764

[11] Ng HH, Robert F, Young RA, Struhl K 2002 Genome-wide location and regulated recruitment of the RSC nucleosome-remodeling complex. Genes Dev 16:806–819. doi:10.1101/gad.978902. 11937489 PMC186327

[12] Kasten M, Szerlong H, Erdjument-Bromage H, Tempst P, Werner M, Cairns BR 2004 Tandem bromodomains in the chromatin remodeler RSC recognize acetylated histone H3 Lys14. EMBO J 23:1348–1359. doi:10.1038/sj.emboj.7600143. 15014446 PMC381415

[13] Soutourina J, Bordas-Le Floch V, Gendrel G, Flores A, Ducrot C, Dumay-Odelot H, Soularue P, Navarro F, Cairns BR, Lefebvre O, Werner M 2006 Rsc4 connects the chromatin remodeler RSC to RNA polymerases. Mol Cell Biol 26:4920–4933. doi:10.1128/MCB.00415-06. 16782880 PMC1489167

[14] Cao Y, Cairns BR, Kornberg RD, Laurent BC 1997 Sfh1p, a component of a novel chromatin-remodeling complex, is required for cell cycle progression. Mol Cell Biol 17:3323–3334. doi:10.1128/mcb.17.6.3323. 9154831 PMC232185

[15] Hsu JM, Huang J, Meluh PB, Laurent BC 2003 The yeast RSC chromatin-remodeling complex is required for kinetochore function in chromosome segregation. Mol Cell Biol 23:3202–3215. doi:10.1128/mcb.23.9.3202-3215.2003. 12697820 PMC153182

[16] Huang J, Hsu JM, Laurent BC 2004 The RSC nucleosome-remodeling complex is required for cohesin’s association with chromosome arms. Mol Cell 13:739–750. doi:10.1016/s1097-2765(04)00103-0. 15023343

[17] Chai B, Huang J, Cairns BR, Laurent BC 2005 Distinct roles for the RSC and Swi/Snf ATP-dependent chromatin remodelers in DNA double-strand break repair. Genes Dev 19:1656–1661. doi:10.1101/gad.1273105. 16024655 PMC1176001

[18] Shim EY, Hong SJ, Oum JH, Yanez Y, Zhang Y, Lee SE 2007 RSC mobilizes nucleosomes to improve accessibility of repair machinery to the damaged chromatin. Mol Cell Biol 27:1602–1613. doi:10.1128/MCB.01956-06. 17178837 PMC1820475

[19] Mao X, Cao F, Nie X, Liu H, Chen J 2006 The Swi/Snf chromatin remodeling complex is essential for hyphal development in *Candida albicans*. FEBS Lett 580:2615–2622. doi:10.1016/j.febslet.2006.04.009. 16647065

[20] Tebbji F, Chen Y, Sellam A, Whiteway M 2017 The genomic landscape of the fungus-specific SWI/SNF complex subunit, Snf6, in *Candida albicans*. mSphere 2:e00497-17. doi:10.1128/mSphere.00497-17. 29152582 PMC5687922

[21] Chu ZJ, Wang YJ, Ying SH, Wang XW, Feng MG 2016 Genome-wide host-pathogen interaction unveiled by transcriptomic response of diamondback moth to fungal infection. PLoS One 11:e0152908. doi:10.1371/journal.pone.0152908. 27043942 PMC4820269

[22] Ortiz-Urquiza A, Keyhani NO 2013 Action on the surface: entomopathogenic fungi versus the insect cuticle. Insects 4:357–374. doi:10.3390/insects4030357. 26462424 PMC4553469

[23] Wang J, Ying SH, Hu Y, Feng MG 2016 Mas5, a homologue of bacterial DnaJ, is indispensable for the host infection and environmental adaptation of a filamentous fungal insect pathogen. Environ Microbiol 18:1037–1047. doi:10.1111/1462-2920.13197. 26714790

[24] Zhu J, Ying SH, Feng MG 2016 The Na^+^/H^+^ antiporter Nhx1 controls vacuolar fusion indispensible for the life cycle *in vitro* and *in vivo* of a fungal insect pathogen. Environ Microbiol 18:3884–3895. doi:10.1111/1462-2920.13359. 27112237

[25] Zhang LB, Tang L, Ying SH, Feng MG 2017 Two eisosome proteins play opposite roles in autophagic control and sustain cell integrity, function and pathogenicity in *Beauveria bassiana*. Environ Microbiol 19:2037–2052. doi:10.1111/1462-2920.13727. 28276124

[26] Cai Q, Wang JJ, Fu B, Ying SH, Feng MG 2018 Gcn5-dependent histone H3 acetylation and gene activity is required for the asexual development and virulence of *Beauveria bassiana*. Environ Microbiol 20:1484–1497. doi:10.1111/1462-2920.14066. 29417710

[27] Tong SM, Zhang AX, Guo CT, Ying SH, Feng MG 2018 Daylight length-dependent translocation of VIVID photoreceptor in cells and its essential role in conidiation and virulence of *Beauveria bassiana*. Environ Microbiol 20:169–185. doi:10.1111/1462-2920.13951. 28967173

[28] Zhang LB, Feng MG 2018 Antioxidant enzymes and their contributions to biological control potential of fungal insect pathogens. Appl Microbiol Biotechnol 102:4995–5004. doi:10.1007/s00253-018-9033-2. 29704043

[29] Tong SM, Feng MG 2019 Insights into regulatory roles of MAPK-cascaded pathways in multiple stress responses and life cycles of insect and nematode mycopathogens. Appl Microbiol Biotechnol 103:577–587. doi:10.1007/s00253-018-9516-1. 30448905

[30] Xiao GH, Ying SH, Zheng P, Wang ZL, Zhang SW, Xie XQ, Shang YF, Zheng HJ, Zhou Y, St Leger RJ, Zhao GP, Wang CS, Feng MG 2012 Genomic perspectives on the evolution of fungal entomopathogenicity in *Beauveria bassiana*. Sci Rep 2:483. doi:10.1038/srep00483. 22761991 PMC3387728

[31] Wösten HAB 2001 Hydrophobins: multipurpose proteins. Annu Rev Microbiol 55:625–646. doi:10.1146/annurev.micro.55.1.625. 11544369

[32] Zhang SZ, Xia YX, Kim B, Keyhani NO 2011 Two hydrophobins are involved in fungal spore coat rodlet layer assembly and each play distinct roles in surface interactions, development and pathogenesis in the entomopathogenic fungus, *Beauveria bassiana*. Mol Microbiol 80:811–826. doi:10.1111/j.1365-2958.2011.07613.x. 21375591

[33] Li F, Shi HQ, Ying SH, Feng MG 2015 WetA and VosA are distinct regulators of conidiation capacity, conidial quality, and biological control potential of a fungal insect pathogen. Appl Microbiol Biotechnol 99:10069–10081. doi:10.1007/s00253-015-6823-7. 26243054

[34] Zhang AX, Mouhoumed AZ, Tong SM, Ying SH, Feng MG 2019 BrlA and AbaA govern virulence-required dimorphic switch, conidiation, and pathogenicity in a fungal insect pathogen. mSystems 4:e00140-19. doi:10.1128/mSystems.00140-19. 31289140 PMC6616149

[35] Etxebeste O, Garzia A, Espeso EA, Ugalde U 2010 *Aspergillus nidulans* asexual development: making the most of cellular modules. Trends Microbiol 18:569–576. doi:10.1016/j.tim.2010.09.007. 21035346

[36] Park HS, Yu JH 2012 Genetic control of asexual sporulation in filamentous fungi. Curr Opin Microbiol 15:669–677. doi:10.1016/j.mib.2012.09.006. 23092920

[37] Otamendi A, Espeso EA, Etxebeste O 2019 Identification and characterization of *Aspergillus nidulans* mutants impaired in asexual development under phosphate stress. Cells 8:1520. doi:10.3390/cells8121520. PMC6952808 31779253

[38] Zhang RG, Andersson CE, Savchenko A, Skarina T, Evdokimova E, Beasley S, Arrowsmith CH, Edwards AM, Joachimiak A, Mowbray SL 2003 Structure of *Escherichia coli* ribose-5-phosphate isomerase: a ubiquitous enzyme of the pentose phosphate pathway and the Calvin cycle. Structure 11:31–42. doi:10.1016/s0969-2126(02)00933-4. 12517338 PMC2792023

[39] Boles E, Hollenberg CP 1997 The molecular genetics of hexose transport in yeasts. FEMS Microbiol Rev 21:85–111. doi:10.1111/j.1574-6976.1997.tb00346.x. 9299703

[40] Henderson PJ, Maiden MC 1990 Homologous sugar transport proteins in *Escherichia coli* and their relatives in both prokaryotes and eukaryotes. Philos Trans R Soc Lond B Biol Sci 326:391–410. doi:10.1098/rstb.1990.0020. 1970645

[41] Bourne Y, Henrissat B 2001 Glycoside hydrolases and glycosyltransferases: families and functional modules. Curr Opin Struct Biol 11:593–600. doi:10.1016/s0959-440x(00)00253-0. 11785761

[42] Pao SS, Paulsen IT, Saier MH Jr 1998 Major facilitator superfamily. Microbiol Mol Biol Rev 62:1–34. doi:10.1128/MMBR.62.1.1-34.1998. 9529885 PMC98904

[43] Walmsley AR, Barrett MP, Bringaud F, Gould GW 1998 Sugar transporters from bacteria, parasites and mammals: structure-activity relationships. Trends Biochem Sci 23:476–481. doi:10.1016/S0968-0004(98)01326-7. 9868370

[44] Maiden MC, Davis EO, Baldwin SA, Moore DC, Henderson PJ 1987 Mammalian and bacterial sugar transport proteins are homologous. Nature 325:641–643. doi:10.1038/325641a0. 3543693

[45] Weber E, Chevallier MR, Jund R 1988 Evolutionary relationship and secondary structure predictions in four transport proteins of *Saccharomyces cerevisiae*. J Mol Evol 27:341–350. doi:10.1007/bf02101197. 3146645

[46] Vandenbol M, Jauniaux JC, Grenson M 1989 Nucleotide sequence of the *Saccharomyces cerevisiae* PUT4 proline-permease-encoding gene: similarities between CAN1, HIP1 and PUT4 permeases. Gene 83:153–159. doi:10.1016/0378-1119(89)90413-7. 2687114

[47] Valls LA, Hunter CP, Rothman JH, Stevens TH 1987 Protein sorting in yeast: the localization determinant of yeast vacuolar carboxypeptidase Y resides in the propeptide. Cell 48:887–897. doi:10.1016/0092-8674(87)90085-7. 3028649

[48] Tate SS, Meister A 1985 Gamma-glutamyl transpeptidase from kidney. Methods Enzymol 113:400–419. doi:10.1016/s0076-6879(85)13053-3. 2868390

[49] Devenish SR, Blunt JW, Gerrard JA 2010 NMR studies uncover alternate substrates for dihydrodipicolinate synthase and suggest that dihydrodipicolinate reductase is also a dehydratase. J Med Chem 53:4808–4812. doi:10.1021/jm100349s. 20503968

[50] Hellman LM, Fried MG 2007 Electrophoretic mobility shift assay (EMSA) for detecting protein-nucleic acid interactions. Nat Protoc 2:1849–1861. doi:10.1038/nprot.2007.249. 17703195 PMC2757439

[51] Gao BJ, Mou YN, Tong SM, Ying SH, Feng MG 7 4 2020 Subtilisin-like Pr1 proteases marking evolution of pathogenicity in a wide-spectrum insect-pathogenic fungus. Virulence doi:10.1080/21505594.2020.1749487. PMC7199741 32253991

[52] Cai Q, Wang ZK, Shao W, Ying SH, Feng MG 2018 Essential role of Rpd3-dependent lysine modification in the growth, development and virulence of *Beauveria bassiana*. Environ Microbiol 20:1590–1606. doi:10.1111/1462-2920.14100. 29575704

[53] Shao W, Cai Q, Tong SM, Ying SH, Feng MG 2019 Rei1-like protein regulates nutritional metabolism and transport required for the asexual cycle *in vitro* and *in vivo* of a fungal insect pathogen. Environ Microbiol 21:2772–2786. doi:10.1111/1462-2920.14616. 30932324

[54] Holder DJ, Kirkland BH, Lewis MW, Keyhani NO 2007 Surface characteristics of the entomopathogenic fungus *Beauveria* (*Cordyceps*) *bassiana*. Microbiology 153:3448–3457. doi:10.1099/mic.0.2007/008524-0. 17906143

[55] Wang JJ, Qiu L, Chu ZJ, Ying SH, Feng MG 2014 The connection of protein *O*-mannosyltransferase family to the biocontrol potential of *Beauveria bassiana*, a fungal entomopathogen. Glycobiology 24:638–648. doi:10.1093/glycob/cwu028. 24727441

