# Supplementary figures and images for: Nuclear Ssr4 Is Required for the In Vitro and In Vivo Asexual Cycles and Global Gene Activity of Beauveria bassiana
Source: mSystems. 2020 Apr 21;5(2):e00677-19. doi: 10.1128/mSystems.00677-19 (PMC7174636; doi:10.1128/mSystems.00677-19)

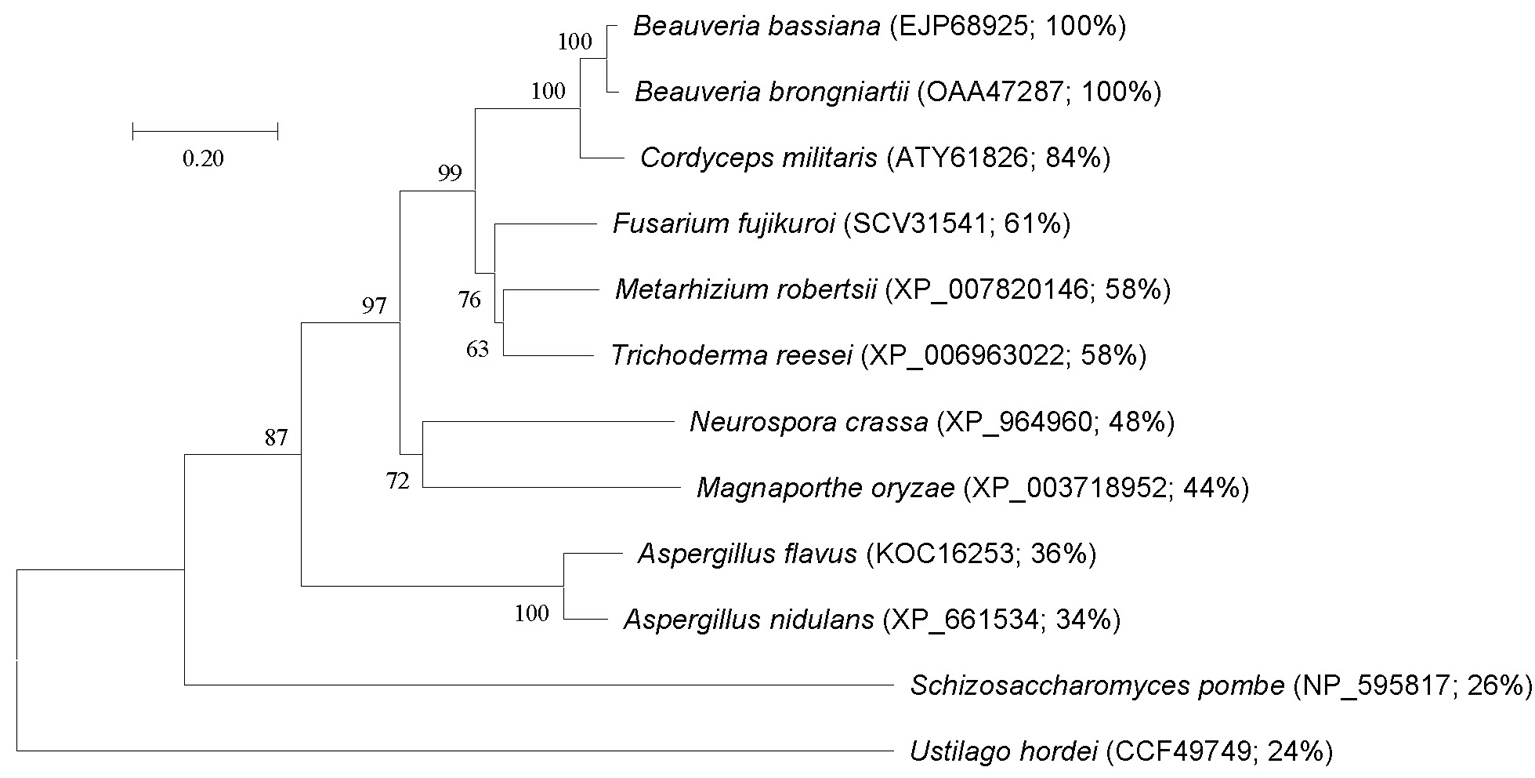

Supplement: FIG S1 [file mSystems.00677-19-sf001.jpg]

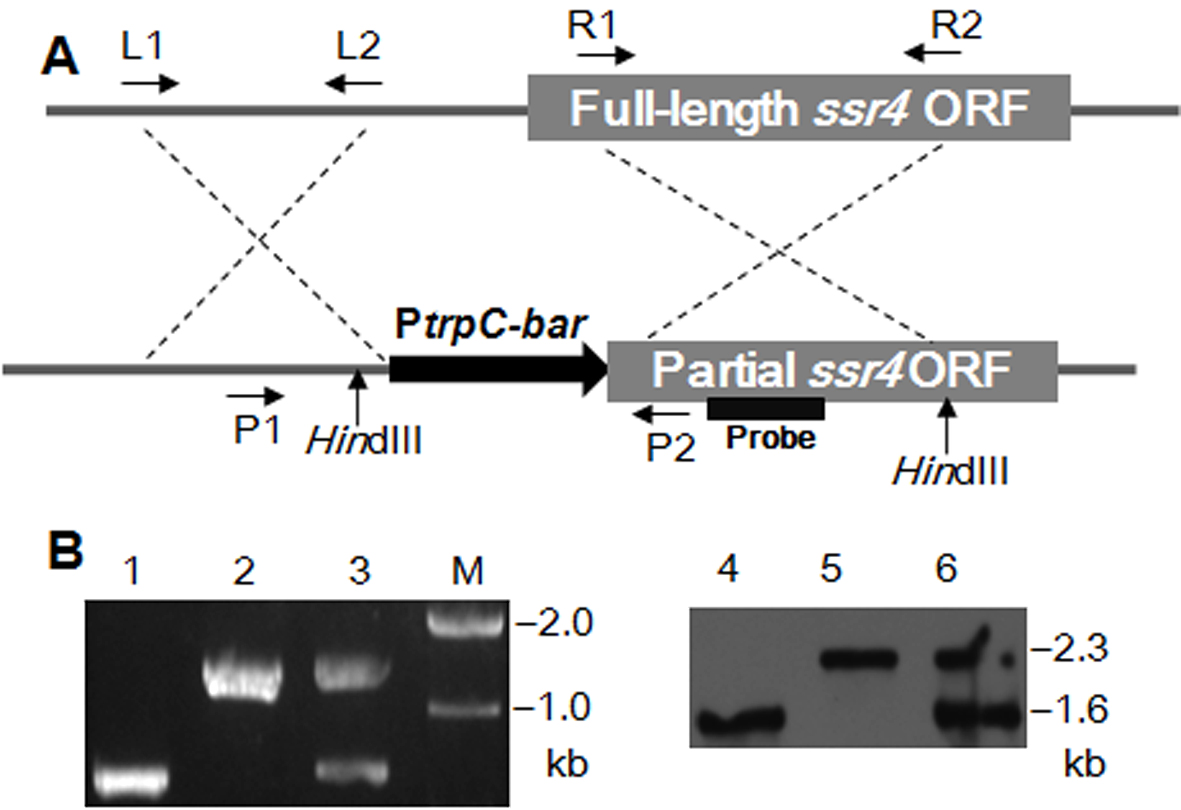

Supplement: FIG S2 [file mSystems.00677-19-sf002.jpg]

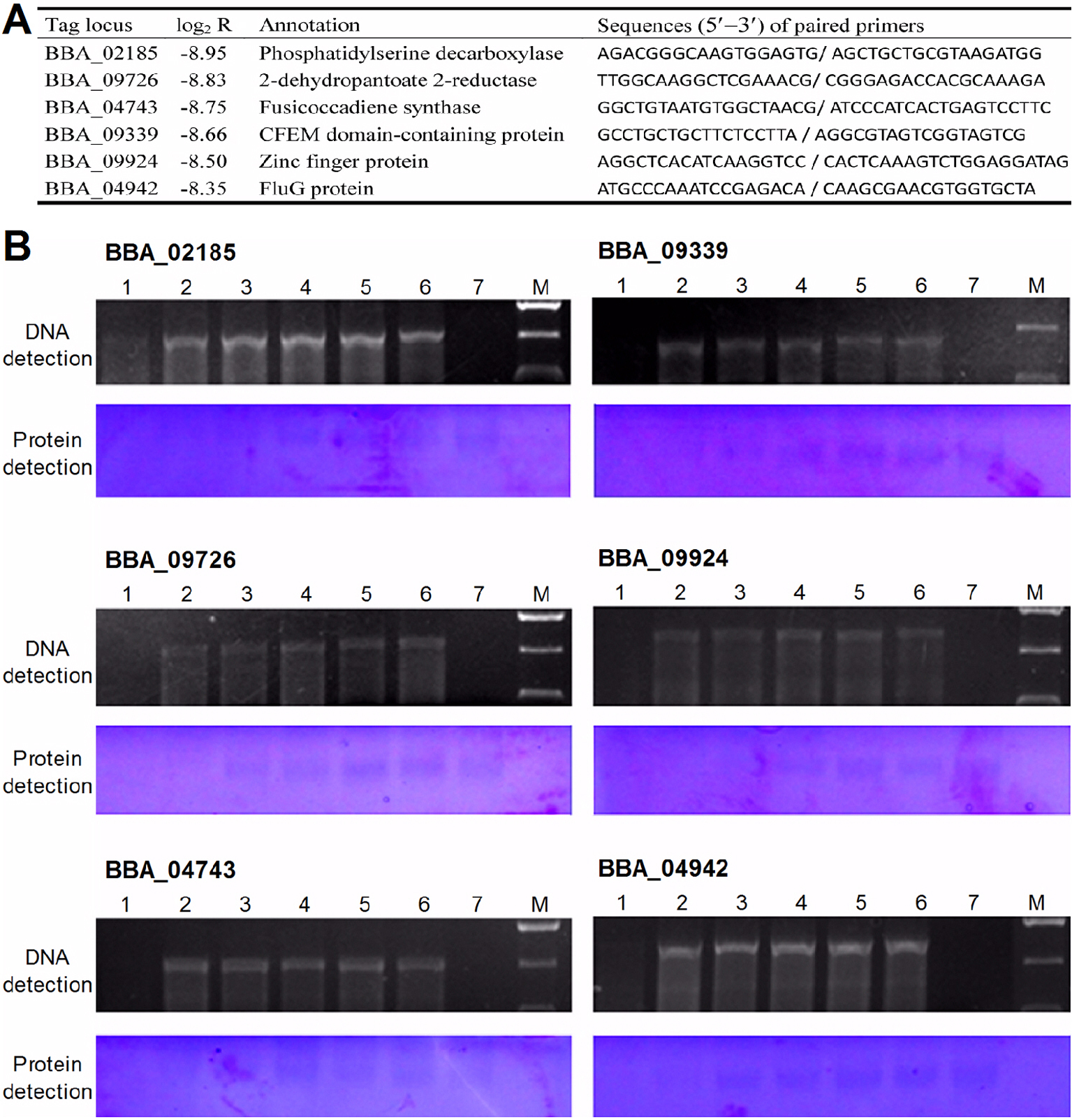

Supplement: FIG S3 [file mSystems.00677-19-sf003.jpg]
