# Supplementary material for: Nuclear Ssr4 Is Required for the In Vitro and In Vivo Asexual Cycles and Global Gene Activity of Beauveria bassiana
Source: mSystems. 2020 Apr 21;5(2):e00677-19. doi: 10.1128/mSystems.00677-19 (PMC7174636; doi:10.1128/mSystems.00677-19)
Supplement: TABLE S1 [file mSystems.00677-19-st001.pdf]

Saccharomyce cerevisiae		*Schizosaccharomyces pombe*		*Beauveria bassiana*		*Botrytis cinerea*		*Magnaporthe oryzae*		*Trichoderma reesei*		*Fusarium fujikuroi*	
SWI/SNF	RSC	SWI/SNF	RSC	SWI/SNF	RSC	SWI/SNF	RSC	SWI/SNF	RSC	SWI/SNF	RSC	SWI/SNF	RSC
**Snf2**/YOR290C	**Sth1**/YIL126W	**Snf22**/SPCC1620.14c	**Snf21**/SPAC1250.01	EJP70213	EJP70213	CCD49650	CCD49650	XP_003717185	XP_003717185	XP_006963468	XP_006963468	SCV30368	SCV30368
**Swi1**/YPL016W		**Sol1**/SPBC30B4.04c											
**Snf5**/YBR289W	**Sfh1**/YLR321C	**Snf5**/SPAC2F7.08c	**Sfh1**/SPCC16A11.14	EJP66839	EJP68944	XP_001546059	XP_001549133	XP_003717270	XP_003710141	XP_006961644	XP_006961537	KLP02132	KLP21684
**Swi3**/YJL176C	**Rsc8**/YFR037C	**Ssr1**/SPAC17G6.10, **Ssr2**/SPAC23H3.10	**Ssr1**/SPAC17G6.10, **Ssr2**/SPAC23H3.10	EJP64113	EJP64113	XP_001558330	XP_001558330	XP_003714717	XP_003714717	ETS05117	ETS05117	KLO80518	KLO80518
**Snf12**/YNR023W	**Rsc6**/YCR052W	**Ssr3**/SPAC23G3.10c	**Ssr3**/SPAC23G3.10c	EJP60960		XP_001548183		XP_003714802		XP_006968138		XP_023433518	
		**Ssr4**/SPBP23A10.05	**Ssr4**/SPBP23A10.05	EJP68925	EJP68925	CCD46304	CCD46304			XP_006963022	XP_006963022	SCV31541	SCV31541
		**Arp42**/SPAC23D3.09	**Arp42**/SPAC23D3.09	EJP70679	EJP70679	EMR84348	EMR84348	XP_003719871	XP_003719871	XP_006966781	XP_006966781	XP_023424811	XP_023424811
**Arp9**/YMR033W	**Arp9**/YMR033W	**Arp9**/SPAC1071.06	**Arp9**/SPAC1071.06	EJP70792	EJP70792	XP_024546234	XP_024546234	XP_003711905	XP_003711905	ETR97804	ETR97804	SCV56192	SCV56192
**Arp7**/YPR034W	**Arp7**/YPR034W			EJP66367	EJP66367	XP_024553767	XP_024553767	XP_003719871	XP_003719871	XP_006961104	XP_006961104	XP_023428783	XP_023428783
**Taf14**/YPL129W		**Tfg**3/SPAC22H12.02		EJP67169		XP_001553002		XP_003712749		ETS03781		KLO79598	
	**Rsc1**/YGR056W, **Rsc2**/YLR357W		**Rsc1**/SPBC4B4.03		EJP63256		CCD34539		ELQ36453		XP_006965753		KLP04580
	**Rsc4**/YKR008W		**Rsc4**/SPBC1734.15		EJP63256		CCD34539		XP_003710047		ETS06884		SCV30368
	**Rsc9**/YML127W		**Rsc9**/SPBC1703.02		EJP63520		XP_001556405		XP_003709290		ETS04559		KLO85510
	**Rsc58**/YLR033W		**Rsc58**/SPAC1F3.07c										
**Swp82**/YFL049W	**Rsc7**/YMR091C	**Snf59**/SPBC26H8.09c	**Rsc7**/SPCC1281.05	EJP66503	EJP66493	CCD50348	XP_024551916	XP_003710227	XP_003710227	XP_006966231	XP_006966376	SCV31457	SCV31457
		**Snf30**/SPAC23G3.07c											
**Rtt102**/YGR275W	**Rtt102**/YGR275W												
**Snf11**/YDR073W													
**Snf6**/YHL025W													
	**Rsc3**/YDR303C												
	**Rsc30**/YHR056C												
	**Ldb7**/YBL006C												
	**Htl1**/YCR020W-B												

* The names (in bold) and NCBI accession codes of all SWI/SNF and RSC components in *S. cerevisiae* and *S. pombe* follow reference 1 (Monahan et al. 2008). The orthologs of those named components in *B. bassaina* and other filamentous fungi are represented by the corresponding NCBI accession codes in the examined fungal genomes.
