# Supplementary material for: Nuclear Ssr4 Is Required for the In Vitro and In Vivo Asexual Cycles and Global Gene Activity of Beauveria bassiana
Source: mSystems. 2020 Apr 21;5(2):e00677-19. doi: 10.1128/mSystems.00677-19 (PMC7174636; doi:10.1128/mSystems.00677-19)
Supplement: TABLE S2 [file mSystems.00677-19-st002.pdf]

Primers	Paired sequences (5′–3′)*	Purpose
cSsr4-F/R	ATGGACGAGCTGTACAAGATGGCGCAACAACCCGAC / CTGCAGGTCGACGGATCCCTATTCTTGCCCCTCGCC	Cloning *ssr4* cDNA (2103 bp) for fusion of *ssr4* to *GFP*
Ssr4up-F/R	TGGGCCCGGCGCGCCGAATTCAGGTTGCCGACAAAGAGGC / TGGCTGCAGGTCGACGGATCCCACGTCCGCGATCCAAGT	Cloning *ssr4* 5′ fragment (1260 bp) for *ssr4* deletion
Ssr4dn-F/R	GACCCATGGCTCGAGTCTAGACGTTTCGATACCCAATAGC / GTGGCTAGCGTTAACACTAGTGGAGGAGGAATCTGATAGCC	Cloning *ssr4* 3′ fragment (1381 bp) for *ssr4* deletion
Ssr4fl-F/R	ggggACCACTTTGTACAAGAAAGCTGGGTCTATTCTTGCCCCTCGCCCGG / ggggACAAGTTTGTACAAAAAAGCAGGCTGCCGTTGCGGTTCTTGTCCT	Cloning full-length *ssr4* (3833 bp) for *ssr4* complementation
pSsr4-F/R	GATGCGATTTCTTCGGTGAG / GCGGAGGTCGTGTTTGTAG	PCR detecting *ssr4*
spSsr4-F/R	CCTCAAGGCTGGCTACATC / CTTCGTCGTCATCCATAACAT	Southern probe of *ssr4*(461 bp)
qSsr4-F/R	AATACATTGCGTCCACAGAA / GAGCCCTCCTTGAACTTG	qPCR detecting *ssr4*
q18S-F/R	TGGTTTCTAGGACCGCCGTAA / CCTTGGCAAATGCTTTCGC	qPCR detecting 18S RNA
qHyd1-F/R	TTCTCAGCGATCTTGATCTT / GCACTTGTTGTCGATTGG	qPCR detecting *hyd1*
qHyd2-F/R	CATGGTGGAAAGGATCTG / ATCTTGGTCGTCTTCTCG	qPCR detecting *hyd2*
qHyd3-F/R	GATATTACAGGCGGCAAT / TGACCACCAGGAATAGAG	qPCR detecting *hyd3*
qHyd4-F/R	CGTCTTTGCTTCTCTCAT / AATTCATCTGCGTTACGA	qPCR detecting *hyd4*
qHyd5-F/R	ATGAAGTTCTTTGCTATCG / CAGACAAGGTTGGAGTAG	qPCR detecting *hyd5*

* Underlined regions denote the restriction enzyme sites for homogeneous recombination of *ssr4* 5′ and 3′ fragments for the deletion of *ssr4* (*Eco*RI/BamHI and *Xba*I/*Spe*I) or the fragments of gateway exchange for *ssr4* complementation. Genomic tag loci of five hydrophobin genes (*hyd1–5*) are BBA_06599, BBA_03015, BBA_00530, BBA_03071 and BBA_02999 under the NCBI accession NZ_ADAH00000000, respectively
